# Predictors of Parental Recall of Newborn Hearing Screening Program in Saudi Arabia

**DOI:** 10.3390/healthcare11091357

**Published:** 2023-05-08

**Authors:** Mohammed A. Almatrafi, Nouf Alsahaf, Abdulrahman Kabli, Lama Maksood, Khawlah Alharbi, Alhanouf Alsharif, Revan A. Mujahed, Abdallah Y. Naser, Hamza M. Assaggaf, Rafat Mosalli, Shahd Alshareef, Emad Salawati

**Affiliations:** 1 Department of Pediatrics, Umm Al-Qura University, Makkah 24382, Saudi Arabia; 2 Medical Intern, College of Medicine, Umm Al-Qura University, Makkah 24382, Saudi Arabia; 3 Department of Otolaryngology, King Abdullah Medical City, Makkah 24246, Saudi Arabia; 4 Department of Applied Pharmaceutical Sciences and Clinical Pharmacy, Faculty of Pharmacy, Isra University, Amman 11622, Jordan; 5 Department of Laboratory Medicine, Faculty of Applied Medical Sciences, Umm Al-Qura University, Makkah 24382, Saudi Arabia; 6 Department of Otolaryngology, King Fahad Hospital, Jeddah 23325, Saudi Arabia; 7 Department of Family Medicine, Faculty of Medicine, King Abdulaziz University, Jeddah 21589, Saudi Arabia

**Keywords:** hearing screen program, parental, newborn, Saudi Arabia, education

## Abstract

Hearing impairment is a prevalent disabling condition among children; all newborns should undergo a universal newborn hearing screening (UNHS). Unfortunately, many newborns who fail the screening test are lost to follow-up. Our study aims to evaluate parents’ perceptions of UNHS and to identify predictors for newborn hearing screening recall in Saudi Arabia. A cross-sectional study involving Saudi parents with 0-to-18-year-old children born in Saudi Arabia was conducted. Descriptive statistics and binary logistic regression were used to describe the participants’ characteristics and to identify UNHS recall predictors. A total of 1533 parents were surveyed. Overall, 29.9% of them recalled a hearing screening at birth, while 22.2% reported no hearing screening, and 47.8% were unable to remember. Only (6.9%) participants reported a failed hearing screening, of which 75.9% recalled a follow-up recommendation. Females, parents aged 30–34 years, consanguineous parents, and parents of newborns who were treated with antibiotics were more likely to recall hearing screening compared to others. This study highlights inadequate awareness of UNHS among parents. Our findings support the need to improve the reporting system of UNHS results and implement educational programs to increase parents’ recall of hearing test results and ensure early follow-ups for neonates with failed test results.

## 1. Introduction

Hearing impairment is a prevalent disabling condition [1]. The World Health Organization (WHO) declares that around 466 million individuals worldwide experience hearing impairment conditions, with children accounting for 34 million of those affected [2]. Globally, the annual cost of untreated hearing impairment is estimated to be around USD 750 billion, including healthcare costs, educational, and social support [2].

Although risk factors for hearing loss may be encountered at various times throughout life, people are most vulnerable to its effects at key life stages, such as the prenatal, perinatal, childhood, and adolescent years, as well as adulthood and older age [3]. The major causes of hearing loss and deafness during the prenatal period include genetic factors, including hereditary and non-hereditary hearing loss as well as intrauterine diseases such as rubella and Cytomegalovirus (CMV) infection. The primary causes of hearing loss and deafness during the perinatal era include birth asphyxia (a lack of oxygen at the moment of delivery), hyperbilirubinemia (severe jaundice in the neonatal period), low birth weight, and various perinatal morbidities and their care [3].

Auditory development in humans begins around the 20th week of gestation and continues for three years [4]. Therefore, early identification and treatment of hearing impairment are crucial to ensure adequate speech and language development and prevent negative consequences on cognition, socio-emotional skills, and academic performance [5,6]. Studies have found that early detection of hearing problems and intervention before six months of age significantly decrease speech and language development delays and improve academic outcomes in affected children [7,8,9,10]. Delays in initiating treatment may result in lower educational and employment levels in adulthood [11].

The National Institute of Health has advised that all newborns should be screened early for hearing impairment, ideally before hospital discharge [12]. Current guidelines recommend that all newborns be screened during their first month, diagnosed, and followed up during the third month of age, and ideally, intervention should be initiated by six months of age [13]. Therefore, the universal newborn hearing screening (UNHS) for hearing impairment has become the standard of care in most countries [14]. The UNHS program screens all newborns, regardless of risk factors, for hearing impairment, allowing for the early referral of newborns with failed screening results for definitive testing and intervention [15,16]. The implementation of the UNHS program has resulted in early detection and intervention for infants with hearing impairment [13].

In 2016, The Saudi Arabian Ministry of Health launched the UNHS program [17]. Despite a slow start, the number of screened newborns increased from 17% to 89% in recent years [18]. Parents and caregivers are critical to any interventional process’s success, including the UNHS program [19]. Newborns with failed hearing screening are referred to an audiologist for further evaluation and diagnostic workup [20]. Unfortunately, many children who fail the hearing screening test are lost to follow-up [21,22]. Parents’ awareness of the program has a tremendous impact on achieving the program’s objectives of early referral to specialists in failed hearing screenings, thereby improving the prognosis and overall well-being of infants with hearing impairments [23,24].

The effects of untreated hearing loss on an individual’s quality of life include communication and speech, cognition, employment and education, social isolation, loneliness, and stigma as well as effects on the economy and society as measured by the years lived with disability (YDLs) and disability-adjusted life years (DALYs) [3].

There are limited studies in the Middle East that have examined the predictors of parental recall of newborn hearing screenings. Exploring parental attitude and recalls of UNHS is very important because it affects the parents’ practices and referral to further testing and diagnosis, ultimately leading to the early identification of newborns with hearing impairments and an improvement in their quality of life if they receive the appropriate treatment and management. Our study aims to evaluate parents’ perceptions of UNHS and to identify predictors for newborn hearing screening recall in Saudi Arabia.

## 2. Materials and Methods

### 2.1. Study Design

A cross-sectional study using a previously validated questionnaire was conducted in Saudi Arabia between April and May of 2021 to evaluate parents’ perceptions of UNHS and to identify predictors for newborn hearing screening recall.

#### 2.1.1. Study Population

Saudi parents with children aged 0 to 18 and born in Saudi Arabia were eligible to participate in the study.

#### 2.1.2. Participants Recruitment

The survey was posted on social media platforms, including WhatsApp, Twitter, and Facebook. A convenience sampling technique was used to select participants who met the study’s inclusion criteria and completed the questionnaire. The questionnaire tool was distributed via social media platforms. The survey asked the parents to provide information about their child’s hearing and neonatal hearing screening. Parents who had multiple children were asked to report on their youngest child regardless of their health status.

#### 2.1.3. Questionnaire Tool

We used a self-administrated online questionnaire which was adapted from a previous study (Supplementary Material Table S1) [22]. The survey was translated into Arabic and validated by three experts in the field using the forward–backwards translation method. This translation method focused more on the meaning and the concept than on a word-by-word translation. The survey tool was piloted on a small group of the targeted population who met our inclusion criteria, and they were asked about the questionnaire tool and whether they found any difficulty in understanding it. They confirmed that it was easy and straightforward. The first part of the questionnaire collected data about the demographics of parents, including age, gender, marital status, level of education, employment, household income, chronic diseases, consanguinity, and family history of hearing impairment. Children information, including the year of birth, region of residence, health condition, and hearing screening related questions were also collected. We determined the outcome measures based on parent recall, which includes receiving a hearing screening, screening test results, and recommendations for follow-up following the screen.

#### 2.1.4. Sample Size

To estimate the sample size, we used the WHO recommendations. The required sample size should be equal to or greater than 385 participants to achieve a 95% confidence interval and a 5% significance level (*p*-value), estimated using OpenEpi VER 3.0 [25].

#### 2.1.5. Statistical Analysis

SPSS version 27 was used to conduct statistical analysis on the collected data. Categorical variables were presented as frequency and percentage. Binary logistic regression analysis was conducted to identify predictors of hearing screening test recall. A 95% confidence interval (*p* ≤ 0.05) was applied to indicate the statistical significance of the results, and a significance level of 5% was assigned.

#### 2.1.6. Ethical Approval

The study was approved by the Biomedical Research Ethics Committee of Umm Al-Qura University (Approval No. HAPO-02-K-012-2022-02-948).

## 3. Results

### 3.1. Participants (Parents) Demographic Characteristics

A total of 1533 participants were involved in this study. More than half of them (65.2%) were females. More than half of them (56.7%) were aged below 40 years. Around 64% of the study participants reported holding a bachelor’s degree. Almost two-thirds of the participants (66.3%) reported their monthly income being 5000 SAR or above. A total of 39.4% reported that one or both parents are suffering from chronic disease(s). Around one-third (29.4%) of the participants reported that they are relatives. Around 12.0% of the participants reported that they or a family member had a hearing impairment that started before the age of 40 years. Table 1 below describes the demographic characteristics of the study participants.

### 3.2. Hearing Screening

When we asked the participants about the status of their child’s conditions at birth, the vast majority of them (84.1%) reported that their child(ren) was born in a hospital, 21.8% were treated for jaundice, 20.4% were in the neonatal intensive care unit (NICU), and 18.3% treated for an infection requiring antibiotics. Less than one-third of the study’s participants (29.9%) confirmed that their child(ren) had a hearing screening test at birth, of which 23.4% reported that their child(ren) passed the test in both ears. For those who have reported problems concerning hearing screening tests at birth (n = 29), 75.9% reported that they were recommended to have a follow-up. The most commonly reported recommendations were evaluation by ENT specialists and obtaining a hearing test within a few months with 31.8% and 22.7%, respectively. Table 2 below describes hearing impairment risk factors in addition to neonatal hearing screening test recall and follow-up recommendation.

### 3.3. Predictors of Hearing Screening Test Recall

Binary logistic regression analysis identified that females, those aged 30–34 years, consanguineous parents, and parents of a newborn treated for an infection requiring antibiotics were more likely to recall hearing screen tests than others (*p* ≤ 0.05). On the other hand, older parents (who are aged 46–55 years) and parents who suffer from any chronic diseases were less likely to recall hearing screen tests compared to others (*p* ≤ 0.05). Table 3 below describes the binary logistic regression analysis.

### 3.4. Hearing Screening Test for Pre-School- and School-Age Children

When the participants were asked whether their child(ren) had a hearing screening test at school, only 12.4% of children received a hearing screening at school, with 77.1% of parents confirming that their child passed the hearing test. The majority (85.7%) of those who failed the hearing test (n = 7) reported following up with their child(ren).

In Saudi Arabia, all hospitals perform a hearing screening test on newborns. National standards for hearing screening tests for older children are being considered. We asked the participants whether they would support national legislation that would require routine hearing screening tests in older children. More than half of them (62.0%) reported that they would recommend this legislation for children aged 2–3 years (preschool). When the participants were asked who they would take him/her to see if they had concerns in the future regarding their child’s hearing, the vast majority of them (80.1%) reported audiologist, refer to Table 4.

## 4. Discussion

For every 1000 newborns, 1 to 2 are born with a permanent hearing loss in either one or both ears [26]; when a baby has spent more than 48 h in critical care, this rises to around 1 in 100. The majority of these infants come from families without a history of severe hearing loss. The development of infants may be profoundly impacted by permanent hearing loss [26].

A hearing screening test determines whether or not a person may have hearing loss. Hearing tests are simple and painless. Hearing tests are painless and safe. The ear probes are gentle, and there are no loud noises. In fact, infants often nod off during screening. It simply takes a few minutes most of the time [27]. All newborns should have a hearing loss screening no later than one month of age. Prior to being discharged from the hospital after delivery, it is helpful if they are tested. It is crucial to have a comprehensive hearing test done as soon as possible, but no later than three months of age, if a newborn does not pass a hearing screening [27]. By the time they are 2 to 2 1/2 years old, children who are at risk for acquired, progressive, or delayed-onset hearing loss should undergo at least one hearing test. Progressive hearing loss is a condition in which the hearing loss worsens with time. Delayed-onset or acquired hearing loss is the term used to describe hearing loss that appears after the infant is born [27]. A comprehensive hearing test should be administered to any child who fails a hearing screening. An audiology evaluation is another name for this examination. The comprehensive hearing test will be performed by an audiologist, a professional qualified to assess hearing. The audiologist will also inquire about the patient’s birth circumstances, family history of hearing loss, and ear infections [27].

The UNHS is now considered the standard of care in many countries worldwide, with a primary goal of rapidly identifying newborns with hearing impairment and facilitating early diagnosis and intervention [13]. Prior research has demonstrated the effective implementation of UNHS programs, with a particular focus on the efficacy of early care intervention; however, parental perception and comprehension of screening results and follow-up instructions have received less attention [28]. Parental acceptance and support are crucial to the success and efficacy of the UNHS program [19]. Our study aims to evaluate parents’ perceptions of UNHS and identify predictors for newborn hearing screening recall in Saudi Arabia.

Our findings indicate almost one-third of participants (29.9%) recalled their child receiving a hearing screening, but nearly half of the participants (47.8%) were unaware of whether their child had a hearing screening at birth, and nearly a quarter of participants (22.2%) report that no screening was performed. These unfortunate findings might indicate poor parental awareness of the negative sequelae of unaddressed hearing impairment. A relatively lower but equally concerning percentage of parents who were uncertain of their child’s hearing screening at birth was observed in a national survey conducted in the United States (47.8% vs. 31.5%). These findings indicate that lack of parental awareness may be a significant impediment to the global benefits of UNHS programs [22].

Our results indicate that 6.9% of the parents who recalled a hearing screening were referred for a failed test in one or both ears. This is relatively higher than the benchmark (4%) recommended by The Joint Committee on Infant Hearing for the final referral rate to diagnostic evaluation [13]. Several studies in Saudi Arabia reported a lower referral rate for formal diagnostic evaluation [29,30]. Alaql estimated that Saudi Arabia’s overall referral rate following neonatal hearing screenings is 0.7% [29]. Another retrospective study conducted in two hospitals in Saudi Arabia reported a referral rate of 1.33% [30]. Since this study reported a 34.9% loss to the system for screening, the accurate referral rate might be higher or lower if the loss to the system cases were included.

Referral rates are probably influenced by the child’s age, protocol design, and the number of screening steps [31]. For instance, using AABR protocols rather than TEOAEs as the initial screening step and increasing the number of screening steps result in a lower referral rate [31]. The discrepancy in the reported referral rates between our study and other similar studies might be justified by using different UNHS protocols and recall bias among study participants.

Despite the improved coverage rate of the UNHS across both developed and developing countries, loss of follow-up (LTF) remains a significant barrier to the benefits of the program [32,33]. The estimated LTF rate in our study (24.14%) was comparable to what has been reported in the literature. A systematic review by Ravi et al. reported the LTF at many centers to be around 20% [34]. In 2019, The CDC reported a close estimate with an overall LTF rate of 27.5% [21]. Several studies have noted a positive correlation between high rates of poor follow-up with educational disparity among parents and lack of parental awareness of UNHS [8,34].

Our study sought the demographic predictors of parental recall of hearing screening. Female participants, parents aged 30–34, and consanguineous parents were more likely to recall hearing screening at birth (35%, 52%, and 32%, respectively). Pynnonen et al. found no significant association between the gender and age of parents and parental recall of hearing screening [22]. Most 30–34-year-olds in our community were married around the time the UNHS program was launched in Saudi Arabia as part of routine newborn health care, so perhaps they reported better recall. These findings could also help us pay more attention to older age group parents and concentrate on raising their awareness of the program’s significance. Similar to Pynnonen et al. study, our study showed no significant association between parental recall of hearing screening and parents’ level of education and monthly income.

A high consanguinity rate of 29.9% among our study participants is not surprising given the high overall rate of consanguineous marriages in Saudi Arabia [35]. Consanguineous marriages increased the risk of hereditary conditions including hereditary hearing impairment. Public awareness and educational activities conducted by the Saudi Arabian Ministry of Health regarding the health problems associated with consanguineous marriage might have contributed to better parental recall of hearing screening.

This study showed that parents of children who were treated for infection with antibiotics during neonatal period were 41% more likely to recall UNHS. Pynnonen et al. reported a similar observation where parents with children who had risk factors, including treatment with antibiotics during neonatal period [22]. Bacterial infection, especially in children, is difficult for parents and makes them more worried than others about their children.

Numerous strategies can be implemented to enhance the effectiveness of UNHS programs, reduce the psychological stress and anxiety associated with failed screening tests, and ensure appropriate follow-up if warranted [22,28,36]. First, increased awareness of the program through structured public health campaigns targeting caregivers and parents [22]. Second, early education about the importance of the program, particularly when initiated before childbirth, has been shown to increase the satisfaction and perception of the program compared to providing education after childbirth [36]. Thirdly, administering hearing screenings in the presence of parents and explaining test results with clear verbal and written recommendations for follow-up may enhance the efficacy of UNHS [22].

The UNHS has assisted in the identification of children with congenital hearing impairment [13]. The school-based hearing screening program is an additional opportunity that may aid in identifying undiagnosed hearing impairment in pre-school- and school-aged children and referral to audiology services for treatment and appropriate rehabilitation [37].

In our study, only 12.4% of parents reported that their children had undergone a school-based hearing screening. In Saudi Arabia, all hospitals conduct neonatal hearing screening as a part of routine newborn care. Nevertheless, screenings for older children are being considered, but they are not yet part of routine care. Hearing screenings are conducted in most Swedish public schools [38]. A study conducted in Sweden revealed that parents have a positive perception and attitude toward school-based hearing screenings [38]. When we asked the participants if they would support mandatory routine hearing screening for older children, more than half (62.0%) reported recommending this legislation for pre-school-aged children (2–3 years). This may indicate an opportunity to establish a formal hearing screening program for preschool and school-aged children in Saudi Arabia and conduct additional research on the program’s effectiveness.

From the prenatal and perinatal stages through old age, prevention of hearing loss is crucial. Nearly 60% of hearing loss in children is caused by preventable factors that may be avoided with the deployment of public health initiatives. Immunization, excellent maternal and childcare habits, genetic counseling, and the recognition and treatment of common ear disorders are a few of them [3]. Effective care of ear disorders and hearing loss depends on early detection. In order to identify hearing loss and associated ear problems in people who are most at risk, thorough screening is necessary. Children of pre-school age and older, as well as newborns and infants, are included. Both clinical and public settings may be used to measure hearing and examine ear problems. With little training and resources, ear disorders and hearing loss may be screened by using tools such as the WHO “hearWHO” app and other tech-based alternatives [3]. To minimize any negative effects, it is crucial to correct hearing loss as soon as it is discovered and in the proper way. The use of hearing technologies, such as hearing aids, cochlear implants, and middle ear implants; the use of sign language and other sensory substitution techniques, such as speech reading, use of print-on-palm or Tadoma methods, signed communication; and rehabilitative therapy to improve perceptual skills and develop communication and linguistic abilities are all options for rehabilitating people with hearing loss [3]. People with hearing loss may access communication and education more easily by using hearing-assistive technology and services such as frequency modulation and loop systems, alerting devices, telecommunication devices, captioning services, and sign language interpretation [3].

Various limitations apply to our research. We are constrained by parental recall bias, and the cross-sectional aspect of the study restricts us from drawing any broad conclusions. Additionally, a self-administered questionnaire via an internet platform may be biased.

## 5. Conclusions

This study highlights inadequate awareness of UNHS among parents. Our findings support the need to improve the reporting system of UNHS results and implement educational programs to increase parents’ recall of hearing test results and ensure early follow-up for neonates who failed test results.

## Figures and Tables

**Table 1:** Participants (parents) demographic characteristics.

Demographic Variable	Frequency	Percentage
Parent’s gender		
Female	1000	65.2%
Male	533	34.8%
Age category		
18–23 years	185	12.1%
24–29 years	237	15.5%
30–34 years	241	15.7%
35–39 years	205	13.4%
40–45 years	311	20.3%
46–50 years	162	10.6%
51–55 years	126	8.2%
56–60 years	44	2.9%
61 years and above	22	1.4%
Parent’s education level		
Secondary school level or lower	364	23.7%
Bachelor’s degree	995	64.9%
Higher education	174	11.4%
Monthly income		
2500 SAR ^1^ or lower	255	16.6%
2500–5000 SAR	260	17.0%
5000–10,000 SAR	448	29.2%
10,000–20,000 SAR	439	28.6%
Above 20,000 SAR	131	8.5%

^1^ SAR: Saudi riyal, SAR equal to 0.27 USD.

**Table 2:** Neonatal hearing screening test.

Variable	Frequency	Percentage
When your child was born, was he/she……..?		
Born in a hospital (yes)	1289	84.1%
Treated for jaundice (yes)	334	21.8%
In the neonatal intensive care unit (NICU) (yes)	313	20.4%
Treated for an infection requiring antibiotics (yes)	281	18.3%
Premature (earlier than 37 weeks gestation) (yes)	153	10.0%
Did your child have a hearing screening test at birth? (n = 1403) *		
Yes	420	29.9%
Not sure	671	47.8%
No	312	22.2%
What were the results? (n = 420)		
Passed in both ears	358	23.4%
Abnormal/failed in one ear	22	5.2%
Abnormal/failed in both ears	7	1.7%
I don’t remember	33	7.9%
Was any follow-up recommended? (n = 29)		
Yes	22	75.9%
What was recommended? (n = 22)		
Evaluation by ear-nose-throat specialist	7	31.8%
Obtain hearing test within a few months	5	22.7%
Observe child for any difficulties	2	9.1%
Evaluation by pediatrician or family doctor	2	9.1%
Obtain hearing test by age 2–3 years	2	9.1%
Hearing screening test in school	2	9.1%
Not sure	2	9.1%

* The number is not equal to the total number of participants due to missing data.

**Table 3:** Binary logistic regression analysis.

Demographic Variable	Odds Ratio	95% Confidence Interval
Parent’s gender		
Male (Reference group)	1.00	
Female	1.35	1.06–1.71 *
Age category		
18–23 years (Reference group)	1.00	
24–29 years	1.34	0.99–1.80
30–34 years	1.52	1.14–2.04 **
35–39 years	0.91	0.65–1.28
40–45 years	1.17	0.89–1.54
46–50 years	0.55	0.36–0.83 **
51–55 years	0.60	0.38–0.95 *
56–60 years	0.99	0.51–1.95
61 years and above	0.59	0.20–1.74
Parent’s education level		
Secondary school level or lower (Reference group)	1.00	
Bachelor’s degree	1.13	0.89–1.43
Higher education	0.89	0.62–1.27
Monthly income		
2500 SAR ^1^ or lower (Reference group)	1.00	
2500–5000 SAR	1.14	0.85–1.53
5000–10,000 SAR	0.93	0.72–1.19
10,000–20,000 SAR	0.86	0.67–1.11
Above 20,000 SAR	1.43	0.98–2.09
Does any of the parents suffer from any chronic diseases? (Yes)	0.75	0.60–9.95 *
Are the two parents relative to each other? (Yes)	1.32	1.03–1.67 *
Have you or any of your family members experienced a hearing loss that began before the age of 40 years? (Yes)	1.02	0.73–1.44
Born in a hospital (Yes)	187	12.2%
Treated for jaundice (Yes)	1.28	0.82–1.98
In the neonatal intensive care unit (NICU) (Yes)	1.16	0.89–1.51
Treated for an infection requiring antibiotics (Yes)	1.03	0.78–1.35
Premature (earlier than 37 weeks gestation) (Yes)	1.41	1.07–1.86 *

* *p* ≤ 0.05; ** *p* ≤ 0.01; ^1^ Saudi Riyal.

**Table 4:** Hearing screening test for pre-school- and school-age children.

Variable	Frequency	Percentage
Has your child ever had a hearing screening test at school? (n = 566)		
Yes	70	12.4%
Not sure	240	42.4%
No	256	45.2%
What were the results?		
Passed	54	77.1%
Failed/abnormal	7	10.0%
Not sure	9	12.9%
Did your child have follow-up after the failed school hearing screening test(s)? (n = 7)		
Yes	6	85.7%
No	1	14.3%
In Saudi Arabia, all hospitals perform a hearing screening test on newborns. National standards for hearing screening tests of older children are being considered. Would you support national legislation that would require routine hearing screening tests in older children at the following ages? (n = 1393)		
2–3 years (preschool)	864	62.0%
6–7 years (1st grade)	282	20.2%
10–11 years (5th grade)	71	5.1%
16–17 years (11th grade)	45	3.2%
I don’t support this legislation	131	9.4%
If you had concerns in the future regarding your child’s hearing, who would you take him/her to see? (n = 1314)		
Audiologist	1052	80.1%
Ear-nose-throat specialist	1037	78.9%
Pediatrician or family doctor	888	67.6%
School personnel involved with screening test	301	22.9%
I don’t know	373	28.4%

## Data Availability

Data will be available by the authors.

## Supplementary Materials

The following supporting information can be downloaded at: https://www.mdpi.com/article/10.3390/healthcare11091357/s1, Table S1: Predictors of parental recall of newborn hearing screening program in Saudi Arabia [22].
