# Supplementary material for: Predictors of Parental Recall of Newborn Hearing Screening Program in Saudi Arabia
Source: Healthcare (Basel). 2023 May 8;11(9):1357. doi: 10.3390/healthcare11091357 (PMC10177918; doi:10.3390/healthcare11091357)
Supplement: Supplementary file 1 [file healthcare-11-01357-s001.zip › healthcare-2265128-supplementary.pdf]

healthcare

MDPI

Table S1. Predictors of Parental Recall of Newborn Hearing Screening Program Questionnaire

Section 1: Parents demographic data
**Q1. Age**
- 18-23 years old<br>- 24-29 years old<br>- 30-34 years old<br>- 35-39 years old<br>- 40-45 years old<br>- 46-50 years old<br>- 51-55 years old<br>- 56-60 years old<br>- 61 years old and above
**Q2. Gender**
- Male<br>- Female
**Q3. Marital status**
- Married<br>- Divorced<br>- Widow
**Q4. Level of education**
- High school or less<br>- Bachelor's Degree<br>- Master's or Ph.D Degree
**Q5. Employment**
- Not currently working<br>- Retired<br>- Healthcare worker
**Q6. Household income**
- Less than 2500 SR<br>- 2500-5000 SR<br>- 5000-10000 SR<br>- 10000-20000 SR<br>- More than 20000 SR
**Q7. Does any of the parents have chronic disease (e.g., Diabetes, Hypertension, Hyperlipidemia, Cardiac disease, Renal disease, Gastrointestinal disease) ?**
- Yes<br>- No
**Q8. Are the parents of the child blood relatives (e.g. first cousins) ?**

- Yes
- No

Q9. Have you or anyone in your family had a hearing loss that began before the age of 40?

- Yes
- No

Section 2: Children information

Q10. Has your child(ren) been diagnosed by a doctor with any of the following health conditions?
(check all that apply, if none, please check 'none of the above')

	Child 1 Age	Child 2 Age	Child 3 Age
Allergies			
Asthma			
Attention deficit/hyperactivity disorder (ADD/ADHD)			
Behavioral disorders			
Birth defects			
Depression			
Hearing problems			
Heart problems			
Lung disease from prematurity			
Overweight or obese			
Seizures			
None of the above			

Q11. What year was your child born?

Q12. What region was your child born in?

- Eastern region
- Western region
- Central region
- Northern region
- Southern region

Section3: Hearing screening

Q13. Have you ever been concerned about any of the following in your child?

	Yes	No
Vision		
Hearing		
Sleep		
Feeding/Nutrition		
Development		

Speech		
Nutrition		
Behavior		
Speech and language		
School performance		
**Q13a. What was the reason for concern with your child's [hearing and /speech/speech and language]?**		
	Yes	No
Lack of response to sounds		
Child not responding to voices or said "what" a lot		
Child talks loudly		
Child's speech is difficult to understand		
Watches TV at loud volume		
School notified me of concern		
Behavioral problems		
Speech delay		
Failed a hearing screening test		
Ear infections		
Family history of hearing loss at young age		
Other		
**Q13b. What was the reason for concern with your child's [behavior/school performance]?**		
	Yes	No
Difficulty paying attention		
Child's speech is difficult to understand		
Child does not understand other people		
Difficult social interactions		
Low grades/not achieving full potential in school		
Tantrums/outbursts or disruptive behavior		
Other		
**Q13c. Did your child ever have tubes (tympanostomy tubes) placed in his/her ears?**		
- Yes		
- No		
- Not sure		
**Q14. When your child was born, was he/she…**		
	Yes	No
Born in a hospital		
In the neonatal intensive care unit (NICU)		
Premature (earlier than 37 weeks gestation)		
Treated for jaundice		
Treated for an infection requiring antibiotics		
**Q15. Did your child have a hearing screening test at birth?**		
- Yes		
- No		

- Not sure
**Q16. What were the results?**
- Passed in both ears<br>- Abnormal/failed in one ear<br>- Abnormal/failed in both ears<br>- I don't remember
**Q17. Was any follow-up recommended?**
- Yes<br>- No<br>- Not sure
**Q18. What was recommended?**
- Observe child for any difficulties<br>- Evaluation by pediatrician or family doctor<br>- Evaluation by ear-nose-throat specialist<br>- Obtain hearing test within a few months<br>- Obtain hearing test by age 2-3 years<br>- Hearing screening test in school<br>- Not sure
**Q19. Has your child ever had a hearing screening test at school?**
- Yes<br>- No<br>- Not sure
**Q20. What were the results?**
- Passed<br>- Failed/abnormal<br>- Not sure
**Q21. Did your child have follow-up after the failed school hearing screening test(s)?**
- Yes<br>- No<br>- Not sure

Q22. What follow-up did your child have?

	Yes	No
Evaluation by pediatrician or family doctor		
Evaluation by ear-nose-throat specialist		
Hearing test at health department		

Hearing test in audiology office
**Q23. When did follow-up occur after the failed school hearing screening test(s)?**
- 0 - 6 months later
- 7 -12 months later
- Over 1 year later
- Unsure
**Q24. What was the result of the follow-up?**
- Hearing is normal without treatment
- Hearing loss, improved without treatment
- Hearing loss, required treatment

Q25. Were any of the following treatments done or accommodations made?

	Yes	No
Medical treatment (antibiotics, other medicine)		
Surgical treatment (ear tubes, other surgery)		
Sitting up front in class		
FM system or assistive device		
Hearing aids		
Continued monitoring with further hearing tests		
Other		

**Q26. Why did your child NOT have follow-up after the failed hearing screening test at school?**
**- My child had a cold/infection at the time of the school screening test**
- Hearing seemed fine to me
- I was told the problem was temporary
- Scheduling problems
- Not sure where to go
- Cost/insurance problems
- Too busy/didn't have time
- Other

Q27. In Saudi Arabia, all hospitals perform a hearing screening test on newborns. National standards for hearing screening tests of older children are being considered.

Would you support national legislation that would <u>require</u> routine hearing screening tests in older children at the following ages?

	Yes	No
2-3 years (preschool)		

6-7 years (1st grade)

10-11 years (5th grade)

16-17 years (11th grade)

**Q28. If routine hearing screening tests were required at the following ages, where would you prefer your child(ren)'s testing occur?**				
	School	Pediatrician/ family doctor's office	Local health department	Audiology or ear-nose-throat specialist office
2-3 years (preschool)				
6-7 years (1st grade)				
10-11 years (5th grade)				
16-17 years (11th grade)				

**Q29. If you had concerns in the future regarding your child's hearing, who would you take him/her to see?**		
	Yes	No
School personnel involved with screening test		
Pediatrician or family doctor		
Ear-nose-throat specialist		
Audiologist		
I don't know		
